# Protocol for the development and validation of a questionnaire to assess concerning behaviours and mental health in individuals with autism spectrum disorders: the Assessment of Concerning Behaviour (ACB) scale

**DOI:** 10.1136/bmjopen-2015-010693

**Published:** 2016-03-22

**Authors:** Paramala Santosh, Joanne Tarver, Felicity Gibbons, Silia Vitoratou, Emily Simonoff

**Affiliations:** 1 Department of Child & Adolescent Psychiatry, Institute of Psychiatry, Psychology and Neuroscience, King's College London, London, UK; 2 Centre for Interventional Paediatric Psychopharmacology and Rare Diseases (CIPPRD), Michael Rutter Centre, Maudsley Hospital, London, UK; 3 Department of Biostatistics, Institute of Psychiatry, Psychology and Neuroscience, King's College London, London, UK

**Keywords:** Autism Spectrum Disorder, Comorbidity, Assessment

## Abstract

**Introduction:**

Co-occurring psychiatric conditions and concerning behaviours are prevalent in individuals with autism spectrum disorders (ASD), and are likely to be detrimental to functioning and long-term outcomes. The cognitive rigidity and deficits in emotional literacy and verbal behaviour that commonly occur in ASD can adversely affect clinicians’ confidence to identify concerning behaviours and mental health problems. There is a need to develop a measure that is tailored towards individuals with ASD, and differentiates between symptoms of psychopathology and core ASD symptoms. Furthermore, it should be modified to capture internalising symptoms that individuals with ASD may find difficult or be unable to verbalise. This protocol describes the intended development and validation of the Assessment of Concerning Behaviour (ACB) scale. The ACB will aim to be a multidimensional measure of concerning behaviours in ASD incorporating self-report, parent/carer, teacher/employer and clinician report versions that can be used across the lifespan and spectrum of intellectual ability.

**Methods and analysis:**

This study will be guided by the methods described in the US Food and Drug Administration Guidance for Industry Patient-reported Outcome Measures. A literature review, cognitive interviews and focus groups with individuals who have experience of working or living with ASDs will be used for item generation. A sample of children and adults with ASD will complete the ACB, in addition to other gold standard measures of concerning behaviour in order to establish the initial psychometric properties of the scale.

**Ethics and dissemination:**

This study has received ethical approval from the NHS Research Ethics Committee: London-Camden and King's Cross (ref: 15/LO/0085). Study findings will be disseminated to healthcare professionals and scientists in the field through publication in peer-reviewed journals and conference presentations.

**Strengths and limitations of this study**

- A strength of this study is that it will follow US Food and Drug Administration guidelines for the development of patient-reported outcome measures. Consequently, it will seek extensive input from service users, parents, families and those with experience of working with autism spectrum disorders.
- All participants will be asked to complete the study questionnaires twice allowing for an assessment of questionnaire sensitivity to change in this initial psychometric evaluation of the instrument.
- A potential limitation of this study is that study questionnaires will be completed in participants’ homes. One may be uncertain about the extent to which measures are completed independently. However, some participants will be completing questionnaires in clinic settings which will allow us to explore levels of support which informants require to complete the questionnaire.

## Introduction

Autism spectrum disorders (ASD) are characterised by restrictive and repetitive interests and behaviours, impaired social and communication skills,^1^ and have a prevalence rate of approximately 1%.^2^ ASD is associated with poor long-term psychosocial impairment^3^ and substantial burden on the individual, their family and caregivers in addition to social and economic burden.^4^ ^5^

Observational studies have revealed high levels of concerning behaviours in ASD that are likely to be of further detriment to long-term functioning and outcomes. Throughout this protocol, the term ‘concerning behaviour’ will be used to refer to any behaviours or emotions that may cause concern for individuals with ASD, their parents/carers or healthcare professionals. Concerning behaviours are likely to have a negative impact on the functioning or well-being of the individual with ASD, their family/carers or wider society. Concerning behaviours could themselves form important treatment targets or could be indicative of the presence of a co-occurring condition requiring further investigation. Among others, concerning behaviours that commonly occur in ASD include aggression, anxiety, phobias, hyperactivity, compulsive behaviour, depression, suicidal ideation or attempted suicide and sleep disorders.^6–11^ While prevalence rates of co-occurring conditions and concerning behaviours in ASD vary among studies, this is likely due to methodological differences in populations assessed (eg, population or clinic-based samples) and measurement instruments used (eg, screening or diagnostic tools). Nonetheless, each of these listed concerns are consistently reported as occurring more frequently in individuals with ASD compared to typically developing populations.^6^

However, many clinicians lack confidence in identifying and evaluating co-occurring conditions in ASD. The presence and heterogeneity of ASD symptoms, intellectual disability and difficulty with communication, can complicate the identification of co-occurring conditions in individuals with ASD who may present with atypical symptoms of psychopathology.^12^ Furthermore, diagnostic overshadowing can lead to symptoms of psychopathology being wrongly attributed as core ASD symptoms^6^ (eg, existing social anxiety may be misinterpreted as lack of social interest).

Early and accurate identification of common concerning behaviours and co-occurring conditions in ASD are paramount, given that symptoms may be more amenable to intervention compared to the core symptoms of ASD.^13–17^ Furthermore, established concerning behaviours and coexisting conditions may be more resistant to treatment;^18^ later diagnosis of co-occurring conditions is related to increased risk of hospitalisation.^19^

### Self-report measures in ASD

Another factor complicating the identification of co-occurring conditions in ASD is the use of self-report measures. In typically developing populations of youth, multi-informant reports incorporating self-report measures are seen as the gold-standard for the assessment of mental well-being. ASD is associated with cognitive rigidity, difficulties with emotion recognition and labelling (alexithymia), and deficits in social cognition; such characteristics are likely to lead to differences in the interpretation of self-report measures that have been validated in typically developing and other clinical populations.

A recent meta-analysis has shown that levels of informant agreement in ASD are similar to levels seen in typically developing populations. Correlations between parent and patient report were moderate (r=0.44, 0.42 and 0.36 for externalising problems, internalising problems, and social skills, respectively;^20^), indicating some agreement, yet unique information being provided by parents and self-reports. It is important that self-report measures are not overlooked when assessing individuals with ASD. However, measures should be modified to the unique needs of individuals with ASD and should be validated in this population.

To the best of our knowledge, no questionnaire currently exists that is targeted towards individuals with ASD, includes self-report versions, and is able to screen for coexisting conditions in ASD individuals. This is possibly because existing measures precede the current understanding that many people with ASD and related developmental disorders also have symptoms and disorders that could benefit from other evidence-based treatment.

### Aim

This protocol outlines the intended development and validation of the ‘Assessment of Concerning Behaviour Scale’ (ACB). The term ‘concerning behaviour‘ was chosen for the title of the questionnaire based on feedback from patient and public involvement panels (PPI panels; including parents of individuals with ASD and individuals with ASD) who disliked the use of the other terms including ‘psychopathology’, ‘abnormal behaviour’, ‘maladaptive behaviour’, ‘symptoms’, ‘challenging behaviour’ and ‘mental health problems’.

The ACB aims to be a multidimensional screening measure of concerning behaviours in ASD. The measure is not intended as a diagnostic tool of co-occurring conditions, but aims to identify the presence of symptoms that may require further, more in-depth assessment. The measure will assess the severity of concerning behaviours to allow for assessment of symptom severity and treatment response over time. It is intended primarily for clinical use, but will also be appropriate for use as an outcome in clinical trials.

The study aims to develop self-report, parent/carer, teacher/employer and clinician report versions that can be used across the lifespan and spectrum of intellectual ability. The aim is to make the resulting questionnaire approximately 40 questions in length that should not take longer than 20 min to complete.

This project is being conducted as part of a larger National Institute for Health Research (NIHR)-funded research project entitled ‘Improving Autism Mental Health’, reference number: RP-PG-1211-20016.

### Methods and analysis

This study will be guided by the methods described in the US Food and Drug Administration (FDA) Guidance for Industry Patient-reported Outcome Measures.^21^ It will involve an iterative process with the following stages: concept identification, concept elicitation, cognitive interviews for instrument refinement and instrument validation. The FDA guideline was chosen as the most appropriate given its emphasis on patient input in order to ensure content validity of the questionnaire. Furthermore, following the FDA guidance will allow the instrument to be used as an outcome measure in clinical trials.

## Phase 1: Qualitative development of the ACB

### Tool review

Initial PPI feedback, collected prior to the start of this project, indicated that a brief, single instrument was needed to capture and assess the multitude of concerning behaviours experienced by individuals with ASD. A brief literature review has been conducted and revealed that no screening tool currently exists that has been developed specifically for use in the ASD population. The research team will draw on extensive clinical experience in the assessment of individuals with ASD, and refer to tools commonly used in clinical practice (eg, the Aberrant Behaviour Checklist, the Developmental Behaviour Checklist) as well as relevant review articles^22^ to identify factors to be considered in the assessment of concerning behaviour in ASD.

### Concept identification

Initially, a literature review will identify concerning behaviours that commonly occur in ASD to be included in the questionnaire. A draft version of the instrument incorporating all these constructs will be created. The draft version will be presented for review to a panel of experts with at least 5 years of clinical experience in ASDs in order to confirm the findings of the literature search, and identify missing constructs. A second draft version will be created based on feedback from the expert panel.

### Concept elicitation

The next stage of the study will involve focus groups with individuals with experience of living or working with ASD. Separate focus groups will be conducted with clinicians and mental health professionals specialising in child and adult ASD and associated conditions, teachers working with children with ASD, parents/carers of children/adults with ASD and children, young people and adults with ASD themselves.

The focus groups will explore which concerning behaviours and feelings affect people with ASD. The groups will follow a semistructured format using open-ended questions to allow participants to discuss their experiences and views. Participants will be asked to discuss behavioural and emotional issues that are additional to the core symptoms of ASD. Focus groups will discuss how these issues present, and how they differ and are distinct from core ASD characteristics. Participants’ views on the initial items developed for the questionnaire (from the literature review and feedback from an expert panel) will also be sought. Participants will be asked about items that they feel are important to be included, as well as items which are irrelevant. Focus groups will be audio recorded, and each group will include approximately 4–8 participants (with the exception of child and adult patient focus groups where a maximum of three patients will be invited in an attempt to avoid overwhelming participants). All participants will be asked to complete a demographic questionnaire during this session.

After completion of the focus groups, a thematic analysis, following the principles of thematic analysis proposed by Braun and Clarke,^23^ will be conducted on the recorded and transcribed data. The concerning behaviours and emotions discussed by participants will be placed into meaningful clusters and groups. Any ‘clusters’ or themes identified in the analysis that were not captured by the literature review and expert panel consultation will be created into items, using language used by participants in the focus groups, where possible.

A third draft of the questionnaire will be created incorporating participants’ feedback. Items generated by the group discussions will be added, while items deemed irrelevant or not important by group members will be deleted. It is expected that approximately 150–200 items will be generated through the literature review and expert feedback, and the focus groups will reduce this number to between 40 and 70 items.

PPI panels recruited for the NIHR grant will also be consulted at this stage, to provide their feedback on this version of the questionnaire.

### Cognitive interviews

All participants taking part in focus groups will be sent a copy of the third version of the questionnaire (via email or post) and invited to take part in follow-up interviews over the telephone. The interviews will follow a semistructured interview format. Techniques, such as ‘thinking aloud’ and verbal probing, will be used to ensure that items are correctly understood, and to identify problems with wording and/or difficulties with response options. In order to reduce participant burden, participants will be encouraged to focus on items which they identified as being particularly difficult or easy to understand, or those which they feel could be reworded more effectively. It is anticipated that each interview will last between 20 and 30 min. Based on this feedback, a β version of the questionnaire will be finalised. This will complete the development stage of the study.

## Phase 2: Psychometric evaluation of the ACB

### Main sample

To establish the initial psychometric properties of the questionnaire, 200 participants will be recruited to complete the ACB, in addition to other standardised measures of concerning behaviour (table 1). The questionnaire battery will also incorporate a treatment report form detailing current medication/therapy/intervention status. The treatment report form will be completed by parent/carers of children with ASD, and adults with ASD will be asked to report on their own treatment. Where adults are unable to report on their own treatment, their parent/carer/relative/partner will be asked to complete the treatment report form. The Social Communication Questionnaire^24^ will be completed by parents/caregivers at study entry for characterisation of the study sample.

**Table 1:** Gold standard instruments to be administered to participants in the main sample during the validation stage of the study

		Administered to							
Measure	Key psychometric information	Child with ASD	Young person with ASD	Adult with ASD	Parent/carer of child with ASD	Parent/carer/partner of adult with ASD	Teacher	Employer	Clinician/researcher
Social Communication Questionnaire (SCQ) Lifetime Version^24^	The SCQ shows strong discrimination between ASD and non-ASD cases (sensitivity 0.88, specificity 0.72^32^). Satisfactory internal reliability of the whole scale has been reported (α=0.90^33^).				**X**	**X**			
Child Behaviour Checklist (CBCL) Parent Report^28^	The Achenbach Manual reports acceptable internal reliability of the scales with α values ranging from α=0.72 for the Anxiety Problems subscale to α=0.97 for the Total Problems subscale.^28^ Eight-day test–retest reliability is acceptable; correlations ranging from r=0.80 for the Anxiety Problems subscale to r=0.94 for the Total Problems subscale.^28^				**X**				
Child Behaviour Checklist (CBCL) Teacher Report^28^	Acceptable internal reliability (α values range from α=0.72 for Somatic Complaints and Thought Problems subscales to α=0.95 for Attention Problems, Rule Breaking Behaviour and Aggressive Behaviour subscales^28^). Sixteen-day test–retest values range from r=0.6 for the Withdrawn/Depressed subscale to r=0.96 for the inattention items.^28^						**X**		
Youth Self-Report (YSR)^28^	Internal reliability values range from α=0.67 for the Anxiety subscale to α=0.95 for the Total Problems scale.^28^ Test–retest (8 days) correlations range from r=0.67 for the Withdrawn/Depressed subscale to r=0.89 for the Externalising Behaviour Subscale.^28^	**X**	**X**						
Adult Behaviour Checklist (ABCL)^29^	Internal reliability coefficients are moderate to strong (α=0.67 for Adaptive Functioning—Friends subscale to α=0.97 for the Total Problems subscale).^29^ Test–retest reliability is acceptable (r=0.73 for the Withdrawn subscale to r=0.94 for Substance Use subscale).^29^					**X**		**X**	
Adult Self-Report (ASR)^29^	Internal reliability estimates are moderate to strong ranging, from α=0.51 for the Adaptive Functioning—Education subscale and Thought Problems subscale to α=0.97 for the Total Problems subscale. Test–retest correlation coefficients range from r=0.71 for the Adaptive Functioning—Job subscale to r=0.99 for the Substance Use—Drugs subscale.^29^			**X**					
Aberrant Behaviour Checklist (ABC)^30^	This scale has 5 subscales with α coefficients ranging between 0.86 for Inappropriate Speech to 0.94 for Hyperactivity.^30^ The 4-week test–retest for all of the subscales are between r=0.96–0.98.^30^				**X**	**X**	**X**	**X**	
The Modified Overt Aggression Scale^34^ ^35^	The inter-rater reliability of this instrument has been assessed in a sample of adults with ID (ICC=0.93;^36^).				**X**	**X**	**X**	**X**	
Clinical Global Impressions Scale (CGI)	The CGI correlates well with well-known efficacy scales.^37^ ^38^ The CGI is used in many treatment trials.^39^								**X**
Home Situations Questionnaire (HSQ)- Modified for ASD^40^	Good internal reliability for both subscales (α=0.89 for Demand Specific subscale and α=0.84 for Socially Inflexible subscale).^40^ Test–retest reliability has been shown to be r=0.57 for the Socially Inflexible subscale, r=0.58 for the Demand Specific subscale and r=0.57 for Total Score.^40^				**X**				
Treatment report form	Non-validated questionnaire developed for this study.			**X**	**X**	X*			
Approximate total administration time (min)		55 (+20 min for ACB)	55 (+20 min for ACB)	72 (+20 min for ACB)	82 (+20 min for ACB)	77 (+20 min for ACB)	55 (+20 min for ACB)	40 (+20 min for ACB)	12 (+20 min for ACB)

*To be completed when adults with ASD are unable to report on their own treatment.

ACB, Assessment of Concerning Behaviour Scale; ASD, Autism Spectrum Disorders; ICC, Intraclass Correlation Coefficient.

Participants’ questionnaire battery will be presented in HealthTracker™, a web-based platform for online completion (www.healthtracker.co.uk) that is currently being used in two EU FP7 projects—the Suicidality: Treatment Occurring in Paediatrics (STOP study; http://www.stop-study.com) and the Managing the Link and Strengthening Transition from Child to Adult Mental Health Care (MILESTONE) project (http://www.milestone-transitionstudy.eu). HealthTracker™ has also been used in a previous questionnaire development and validation study.^25^ The web-based questionnaires will follow the principles of data protection, security levels for health data, have an audit trail and Good Clinical Practice compliance. Participants will be given a unique ID number and log-in information, and will be asked to complete the questionnaires independently. All participants, including those with literacy issues, will have the option of going through the questionnaires over the phone with a member of the research team. Participants will also be able to complete paper versions of the questionnaires if requested. Questionnaire completion should not take longer than approximately 60 min for adults and 40 min for children.

Participant medical records will be accessed to validate the ACB against details of diagnoses obtained from patient case notes as well as against Development and Well-being Assessment (DAWBA^26^) diagnoses, IQ and treatment/medication status if they are available in case notes. Consent will be obtained to access medical notes. For children recruited via schools, the research team will also request details about IQ, educational attainment and diagnoses from school records. Parental consent will be obtained to do this. Where measures of IQ are not available from participant medical/school records, a brief measure of IQ will be collected by the research team (see table 1).

All participants will be asked to complete the questionnaire battery twice: at study entry and again between 4 and 6 months after initial completion. This is to allow for analysis of the stability of the questionnaire. Based on clinical experience, 4–6 months was chosen as an appropriate period of time in which one would anticipate change in symptoms (eg, in relation to treatment response). Information on treatment, diagnoses and other relevant information will be gleaned from medical records in order to estimate when change of scores would be expected.

Approximately 50 participants, along with their parent/carer/relative/partner, teacher/employer and clinician will be asked to complete the ACB and gold standard questionnaires (see table 1) again, 1 week after their initial completion, to provide a measure of test–retest reliability. However, interim analyses will be conducted after 25 participants have completed the measure at 1 week. If a statistically significant correlation is evident, no further participants will be asked to complete measures again after 1 week. Since some of the items measured in the ACB are likely to be related to changeable constructs, a priori decisions will be made about items in the ACB that are expected to remain stable over 1 week.

### Augmented sample for factor analysis

In order to permit a factor analysis of the ACB, we will then aim to augment the main sample with a further 150 children and 150 adults with ASD in addition to their parent/carer/relative/partner, teacher/employer and clinician, who will complete the ACB only. Attempts will be made to ensure that the augmented sample is comparable with the main sample on age, gender, IQ and recruitment source. Participants in the augmented sample will be asked to complete the ACB, in addition to providing demographic information. Similar to the main sample, the ACB will be presented to participants in HealthTracker™. Participants will only be required to complete the ACB once, after which, their involvement in the study will end.

#### Recruitment

Information sheets (and age-appropriate information sheets where relevant) will be provided for all participants. Written informed consent and assent (where appropriate) will be obtained from all participants taking part in either stage of the research project. The purpose of the study and issues surrounding consent and confidentiality will be outlined to participants before consent is obtained.

Where the research team has doubts about a person's capacity to consent, a capacity checklist will be completed with the participant. If a participant is unable to provide informed consent, consent for their participation will be gained from their legal guardian or a personal consultee, and assent will also be sought from the participant.

##### Phase 1 recruitment

Children (7–12 years), young people (13–18 years) and adults with ASD, parent/caregivers of individuals with ASD, and teachers and clinicians who work with individuals with ASD will be recruited. Owing to the group-based nature of the focus groups, adults lacking capacity to consent will not be able to partake in focus groups and will be excluded. Separate focus groups will be conducted with each group (children with ASD, young people with ASD, adults with ASD, parents/carers, teachers and clinicians). Approximately 3–8 participants will take part in each focus group. No more than 60 participants in total will take part in the instrument development stage of the research.

Participants with ASD, and parents of individuals with ASD, will be recruited from clinics within participating trusts that see people with ASD and related developmental disorders. The research team or clinicians involved in the patients’ usual care will invite individuals with ASD and parents of children with ASD to take part. Attempts will be made to recruit individuals with a range of concerning behaviours and intellectual abilities, through consultation with the clinician involved in the patient's usual care, and through recruitment from clinics specialising in different disorders associated with ASD. Furthermore, the sample will also consist of individuals recruited from non-clinical sources in order to include participants who have received a diagnosis but are not currently accessing clinical services. This will include recruitment from schools catering to a range of intellectual abilities and from community sources, such as support groups.

Clinicians who work with ASD in participating trusts will be invited to take part in focus groups and follow-up cognitive interviews. Teachers will be invited by contacting local autism-specialist schools in the London area via invitation letter to the head teacher.

Inclusion criteria

- Children (aged 7+ years), young people and adults with ASD (via clinician/researcher invite).
- Parents/carers of children with ASD.
- Teachers working within autism specialist schools for children and young people in the London area.
- Clinicians who work within healthcare settings in participating trusts that see children and/or adults with ASD and associated developmental conditions.

Exclusion criteria

- Participants who do not have a reasonable level of English; a reasonable level of English, will be required to engage in the focus group/cognitive interviews.
- Individuals with ASD who are not verbal will not be included in focus groups/interviews. However, carers, teachers and parents of that person may still take part in their respective groups if they wish.
- Adults lacking capacity to consent will not be included in focus groups/interviews.
- If children, young people, or adults with ASD are not able to (or expected to not be able to) partake in the focus groups, they will be excluded from the instrument development stage. Discussions with clinicians involved in the patient's usual care/caregivers/the patient themselves prior to consent will be used to explore the individual's ability to partake in focus groups, and the levels of distress it would potentially cause them.

##### Phase 2 recruitment

###### Main sample

Individuals with ASD (children, young people and adults) and their parents/caregivers/relatives/partners, teachers/employers and clinicians will be invited to take part (see below for inclusion criteria). Individuals with ASD will be recruited via clinician/researcher invite as well as via poster advertisements within healthcare and community settings, social media and websites for ASD support groups. Individuals with ASD will also be recruited from autism-specific schools and residential settings. Researchers will primarily be recruiting participants via clinics within participating trusts; the aim is to recruit approximately 75% via this method. This is to ensure access to medical notes for as many participants as possible. However, participants will not be excluded if they are not currently under the care of participating trusts. Parental consent will be obtained to request details about the child's IQ, educational attainment and diagnosis from school records.

Parents of children with ASD will be asked to provide details of their child's teacher and clinician, and will provide consent for the research team to contact them and invite them to complete the study questionnaires. Adults with ASD will be asked to provide details of another person who knows them well (either a parent/caregiver/relative/partner) who would be able to complete a questionnaire about them. Adults with ASD will also be asked to provide contact details for their clinician and their employer (if applicable and appropriate).

###### Augmented sample

A similar recruitment approach will be adopted for the augmented sample to ensure comparability with the main sample on demographic characteristics and source of recruitment.

Inclusion criteria

- Children (aged 7+ years), young people and adults with ASD.
- Parents/carers, teachers and clinicians of individuals with ASD.
- Partners/caregivers/relatives/partners and employers of adult individuals with ASD, when parents and teachers are not relevant or appropriate.
- Adults lacking capacity to consent will be included in the instrument validation stage of the study as long as informed consent for their participation is obtained from a legal guardian/personal consultee.

Exclusion criteria

- If researchers have doubts about a participant's capacity to consent after completing a capacity checklist, and an appropriate personal consultee cannot be identified, they will not be consented into the study. Doubts regarding capacity to consent will be discussed with the CI.
- If children, young people, or adults with ASD are not able to (or expected to not be able to) complete questionnaires, they will be excluded from the study. Discussions with clinicians involved in the patients’ usual care/caregivers/the patient themselves prior to consent will be used to explore the individual's ability to complete questionnaires, and the levels of distress it would potentially cause them.
- Participants who do not have a reasonable level of English will be excluded from the instrument validation stage of the study. This is because a reasonable level will be required to complete questionnaires which will only be available in English at the validation stage.

##### Phase 2 sample size

###### Main sample

For this stage of the study, we will aim to recruit 200 people with ASD (approximately 100 adults and 100 children), in addition to their parent/carer/partner, teacher/employer and clinician, to complete the ACB and gold standard measures for analysis of reliability and validity of the ACB (see table 1). In a population cohort of people with ASD, the approximate IQ distribution was as follows: 15% had IQ<50, 40% had 50<IQ<70, 40% had an average IQ (70<IQ<115) and 5% had an above average IQ (IQ>115).^18^ We will aim to recruit across the spectrum of IQ in similar proportions to this population sample to provide a representation of a ‘typical’ ASD population, and ensure generalisability of findings. Attempts will be made to obtain estimates of IQ for individuals with ASD from school/medical notes. However, where this is not available, the research team will collect a brief measure of IQ through completing the Wechsler Abbreviated Scale of Intelligence^27^ during brief home visits to participant homes. All parents, caregivers, relatives and partners will be asked to provide an estimate of the cognitive functional age of the individual with ASD on their demographic questionnaire.

#### Augmented sample

In order to permit a factor analysis of the items with the measure, we will augment the main sample with a further 150 children with ASD and 150 adults with ASD who will complete the ACB only. Where possible, parents/carers, teachers/employers and clinicians will also be recruited into this augmented sample, and asked to complete the appropriate version of the ACB. This augmented sample will be combined with the main sample to ensure that there will be at least six respondents per item for both the child and adult versions of the ACB for the factor analysis.

### Analysis strategy

#### Instrument development

Data collected during focus groups will be recorded and transcribed verbatim. Thematic analysis and content analysis will be used to organise focus group data into meaningful themes. The NVivo software package will be used to manage qualitative data generated from participant focus groups. The data analysis will follow the six steps to thematic analysis proposed by Braun and Clarke.^23^

#### Instrument validation

Quantitative data will be analysed using the SPSS and the MPlus statistics packages.

##### Reliability

###### Internal consistency

Cronbach's α values will be calculated for the total scale and subscales identified by factor analysis. α Values of 0.80, or higher, are commonly accepted as evidence of adequate internal reliability. An ‘if item deleted’ analysis will be conducted to identify whether any items should be dropped from the scale.

###### Test–retest reliability

To assess test–retest reliability, intraclass correlation coefficients will be calculated on subscale and total scores which are expected to remain stable. As this is an exploratory study, weighted Cohen's κ values will be also calculated to assess test–retest reliability at the item level. Coefficients will be established separately for children and adults, and also for parent/carer/relative/partner, teacher/employer and clinician reports on samples of approximately 50 questionnaires completed approximately 1 week after initial completion. For scores that are expected to remain stable, we anticipate a high intraclass correlation of 0.80, for which a sample of 37 would provide a 95% CI of width 0.2. Intraclass correlation coefficients will also be calculated to assess the longer term stability of the measure 4–6 months after initial completion of the questionnaire.

###### Inter-rater reliability

Intraclass correlations will be used to assess inter-rater agreement between pairs of scores provided by two different raters (eg, self-report vs parent/carer; parent/carer vs teacher). Coefficients will be calculated at the subscale and total score levels. As this is an exploratory study, weighted Cohen's κ values will also be calculated to assess inter-rater reliability at the item level.

##### Validity

Scale comparisons will be used to investigate the concurrent convergent validity of the ACB. Pearson correlation coefficients will be calculated to explore the association between ACB scores and other measures collected that are commonly used in clinical practice (table 1; subscale scores will be investigated where appropriate).

Receiver operating characteristic (ROC) analyses will be conducted to evaluate the discriminative power of the instrument. Currently, there is no existing gold-standard instrument for the identification of concerning behaviour in ASD. Therefore, ROC analyses will be conducted using scores from the Achenbach suite of measures (Child Behaviour Checklist;^28^ Youth Self-Report;^28^ Adult Behaviour Checklist;^29^ Adult Self-Report^29^), and the Aberrant Behaviour Checklist^30^ collected as part of the validation phase of this study (see table 1). Where data are available, ROC analyses will also be conducted according to the presence or absence of clinical and/or the structured DAWBA diagnosis taken from the patient's medical notes. For clinical cut-offs, we are able to estimate the ROC against the structured psychiatric assessment with a 95% CIs of ±0.12 (assuming a false-positive rate of 20% and true-positive rate of 28%^31^). In addition, independent samples t tests will be performed with grouping variable DAWBA and/or clinical diagnoses (coded 1 for positive and 0 for negative diagnosis) to assess differences in ACB scoring and the ability of the ACB to distinguish between different groups of patients.

###### Factor analysis

To identify meaningful item clusters, exploratory factor analysis for ordinal data will be used for exploratory analyses of the item pool. If required (eg, in the case of Likert scales having fewer than five response options, substantial floor/ceiling effects, highly skewed items, data not missing at random), item factor analysis will be conducted in MPlus (latent trait model) to avoid biased parameter estimates.

## Ethics and dissemination

This study has received ethical approval from the NHS Research Ethics Committee: London-Camden and King's Cross (ref: 15/LO/0085) and is currently recruiting participants. The aim of the study is to develop and validate an instrument to assess mental health and concerning behaviour in ASD. The study output will provide a clinically relevant tool, predominantly for use with individuals with ASD. Future research will require the recruitment of a second sample of participants in order for Confirmatory Factor Analysis to be conducted on the proposed factor structure resulting from this study. It is also hoped the instrument will be effective as an outcome measure for use in ASD intervention research. If the instrument is found to be a valid measure of concerning behaviour in ASD, future research will also need to assess the sensitivity of the instrument to change following intervention in a randomised controlled trial. The findings of this study will be disseminated to healthcare professionals and scientists in the field through publication in peer-reviewed journals and conference presentations.
